# Effects of Selenylation Modification on Antioxidative Activities of *Schisandra chinensis* Polysaccharide

**DOI:** 10.1371/journal.pone.0134363

**Published:** 2015-07-31

**Authors:** Chanjuan Yue, Jin Chen, Ranran Hou, Jie Liu, Xiuping Li, Zhenzhen Gao, Cui Liu, Deyun Wang, Yu Lu, Hongquan Li, Yuanliang Hu

**Affiliations:** 1 Institute of Traditional Chinese Veterinary Medicine, College of Veterinary Medicine, Nanjing Agricultural University, Nanjing, 210095, PR China; 2 National Research Center of Veterinary Biological Engineering and Technology, Jiangsu Academy of Agricultural Sciences, Nanjing, 210014, PR China; 3 College of Animal Science and Veterinary Medicine, Shanxi Agricultural University, Taigu, Shanxi, 030801, PR China; University of Calcutta, INDIA

## Abstract

The selenylation modification of *Schisandra chinensis* polysaccharide (SCP) was conducted by the HNO_3_–Na_2_SeO_3_ method respectively under nine conditions according to L_9_(3^4^) orthogonal design. Nine selenizing SCPs, sSCP_1_–sSCP_9_, were obtained, and their antioxidant activities were compared. In vitro test, the free radical-scavenging rates of nine sSCPs were determined for DPPH., .OH and ABTS^+^. sSCP_1_ presented the most significant effect, and could inhibit the nonenzymatic protein glycation. In vivo test, 14-day-old chickens were injected respectively with sSCP_1_ and SCP, the serum contents of CAT, SOD and MDA were determined. The result showed that as compared with the SCP group, the SOD and CAT activities were significantly or numerically raised and MDA content was significantly or numerically lowered in the sSCP_1_ group. These results indicate that selenylation modification can significantly enhance the antioxidant and antiglycative activity of SCP in vitro or in vivo. sSCP_1_ possesses the best efficacy and its modification conditions can be as optimal modification conditions that were 200 mg of Na_2_SeO_3_ for 500 mg of SCP, reaction temperature of 50°C and reaction time of 6 h.

## Introduction

In normal cellular metabolism, several reactive oxygen species (ROS) can be generated, such as hydrogen peroxide (H_2_O_2_), superoxide anion (O^-2^·), hydroxyl radicals (·OH) and so on. These ROS can be initiatively scavenged by antioxidant enzymes including superoxide dismutase (SOD), catalase (CAT), glutathione peroxidase, glutathione S epoxide transferase, etc., thus keep the dynamic balance. However, when ROS are excessively generated beyond the scavenging ability or antioxidant function of organism declined, they will cause oxidative damage of DNA, proteins, lipids and small molecules substance and lead to many diseases and age-related anaplasia [1]. At this time it is necessary to supplement a variety of exogenous antioxidant substances from food or drug [2].

Selenium, an essential micronutrient for human and animals, plays an important role in many physiological processes. It also is a key constituent of selenoproteins. Selenium plays important roles especially in anti-oxidant and immunoenhancement [3, 4]. The recent researches have showed that many Chinese medicinal polysaccharides can not only inhibit the formnation of free radicals or directly scavenge free radicals, but also enhance the antioxidant function of organism [5, 6].

Selenium polysaccharide is one of organoselenium compounds. It possesses higher antioxidative activity and safety as compared with inorganic selenium [7]. For example, selenized *artemisia* sphaerocephala polysaccharide showed greater scavenging ability for hydroxyl and superoxide radical as compared with *artemisia* sphaerocephala polysaccharide [8]. The intracellular selenium polysaccharide from *cordyceps* sinensis could increase SOD and GSH-Px activities, reduce the MDA content and scavenge hydroxyl radical in mice [9].

Natural selenium polysaccharides exist in some plants or microorganisms, but their contents are lower even though the plants are grown in the high-selenium area so that the need of selenium polysaccharide is far from being met. Therefore, biotransformation or chemical modification methods were investigated in order to obtain more selenium polysaccharide or selenizing polysaccharide [10].

*Schisandra chinensis* is a common herbal medicine with sedative and tonic actions [11]. It and its extraction can significantly inhibit or directly scavenge free radicals and enhance the antioxidant function in animal [12]. *Schisandra chinensis* polysaccharide (SCP), an important active component of SC, possesses many pharmacological actions such as antioxidant [13], antitumor and immunoregulation [14]. *Schisandra chinensis* contains a water-soluble polysaccharide with molecular weight of 5.3 × 10^3^ Da. It is composed of Man, Glu and Gal, and has a triple helix stereo-configuration and a backbone of →1)-_D_-Man-(6→, →1)- _D_-Manp-(2→,→1)- _D_-Glup(4→, →1)- _D_-Glup-(6→, →1)- _D_-Galp-(4→, →1)- _D_-Galp-(4,6→ and →1)- _D_-Manp-(3,6→, with Man, Glu and Gal, which are distributed in branched chains [15].

In this study, SCP was extracted, purified and modified in selenylation by HNO_3_-Na_2_SeO_3_ method according to L_9_(3^4^) orthogonal design of three factors (usage amount of sodium selenite, the reaction temperature, and reaction time). Nine selenizing SCPs, sSCP_1_–sSCP_9_, were obtained. Their antioxidant activities in vitro were compared by free radical-scavenging test. sSCP_1_ was picked out and its antiglycative activity in vitro and effects on serum contents of CAT, SOD and MDA in chicken were determined. The purpose of this research was to verify the potential of selenylation modification to enhance the antioxidant activity of SCP, screen the best sSCP and optimal selenylation modification condition and offer theoretical evidence for the development of polysaccharide antioxidants.

## Materials and Methods

### 2.1 Drug and reagents

*Schisandra chinensis* was purchased from Xuzhou Pengzu Chinese Herbal Medicine Co., Ltd. Sodium selenite (Na_2_SeO_3_) and nitric acid (HNO_3_) were bought from Shanghai Lingfeng Chemical Reagent Ltd. and Na_2_SeO_3_ dissolved into 0.05 g·mL^-1^ with ultrapure water.

Standard selenium stored solution at 100 μg·mL^-1^ supplied by National standard substance research center, was accurately diluted into 1 μg·mL^-1^. Potassium bromide and potassium persulfate were purchased from Sinopharm Chemical Reagent Co., Ltd. 1,1-diphenyl-2-picrylhydrazyl (DPPH) was purchased from Tokyo Chemical Industry was dissolved with dehydrated alcohol into 10 μg·mL^-1^. 2,2`-Azino-bis-(3- ethylbenzothiazoline-6-sulfonic acid) diammonium salt (ABTS) and Girard’s reagent T were bought from Sigma Aldrich Co., Ltd. ABTS was dissolved into 7 mM with potassium persulfate of 2.45 mM lucifugal reacting for 12–16 h at room temperature. Before use, the solution was diluted with phosphate buffered saline (PBS, 10 mM, pH7.4) into the concentration with absorbance of 0.700±0.02 at a wavelength of 734 nm. Phosphate buffered saline (PBS, pH 7.4, 200 mM) was added with 0.02% sodium azide. Nitro blue tetrozolium (NBT) was dissolved with sodium carbonate buffer (100 mM, pH 10.35) and 2-Deoxy-D-Ribose were purchased from Shanghai Ryon Biological Technology Co., Ltd. Thiobarbituric acid (TBA) was dissolved with 50 mM NaOH sulotion. The kits of CAT, SOD and MDA were bought from Nanjing Jiancheng Bioengineering Institute. Edathamil disodium, trichloride ferric, hydrochloric acid, hydrogen peroxide and other reagents were analytical grade.

### 2.2 Preparation of SCP

SC was soaked in 95% ethyl alcohol for one night, reflowed thrice at 100°C to remove fat, dried in air and decocted thrice with water of 15-fold volume (w/v), each for 3 h after ultrasonic treatment for 30 min. The filtrate was concentrated and centrifugated at 3500 rpm for 20 min. The supernatant was added slowly with 95% alcohol up to 80% of ethanol content. After standing overnight, the precipitate was lyophilized in vacuum freeze-drying machine (Scientz-12N, Ningbo Xinzhi Biotech Co., Ltd.) [16]. The polysaccharide was deproteinized by trichloroacetic acid method to obtained purified SCP finally [14]. Its carbohydrate content was 67.82% measured by phenol-sulfuric acid method [17].

### 2.3 Selenylation modification of SCP

HNO_3_-Na_2_SeO_3_ method was applied in selenylation modification [18]. The modification conditions were optimized by L_9_ (3^4^) orthogonal test of three factors respectively at three levels (Table 1). According to our previous study [19, 20], nine parts of SCP (0.5 g) were respectively dissolved with 50 mL of 5% HNO_3_ solution and added into three-necked flask, the sodium selenite solution was added to intiate the reaction. The reaction liquid was cooled to room temperature after the reaction finished, its pH value was adjusted to 5.5 with 5.6% sodium bicarbonate solution, and the supernatant was dialyzed against distilled water to wipe out free sodium selenite. The result solutions were concentrated and lyophilized. Nine selenizing sSCPs, named sSCP_1_–sSCP_9_, were obtained.

**Table 1:** The modification conditions, yields, contents of selenium and carbohydrate of sSCPs.

sSCPs	A Temperature (°C)	B Na_2_SeO_3_ (mg)	C Time (h)	Yeild (%)	Selenium content (mg·g^-1^)	Carbohydrate content
sSCP_1_	50	200	6	41.23	5.41	56.83
sSCP_2_	50	300	8	54.87	5.24	51.23
sSCP_3_	50	400	10	53.71	10.35	50.28
sSCP_4_	70	200	8	49.15	5.57	55.37
sSCP_5_	70	300	10	53.98	3.82	52.89
sSCP_6_	70	400	6	51.32	3.81	48.81
sSCP_7_	90	200	10	38.11	4.01	50.11
sSCP_8_	90	300	6	32.65	5.03	47.25
sSCP_9_	90	400	8	34.72	5.19	50.84

### 2.4 Identification of sSCP

#### 2.4.1 Selenium content assay

The atomic fluorescence spectrometry was used with atomic fluorescence spectrometer (Model AFS-930, Beijing Jitian instrument Co., Ltd.) [19]. The concentrations of standard curve were set at 0.0, 2.5, 5.0, 10.0, 15.0 and 20.0 ng·mL^-1^, their fluorescence intensity were detected. The standard curve was drawn taking the fluorescence intensity as ordinate and selenium mass concentration as abscissa. The linear regression equations of standard curve was *I* = 125.7658 + 9.9976.

sSCP (0.20 g) was dissolved in 10 mL of ultrapure water. 0.5 mL of sSCP solution was added into 250 mL narrow neck flask with tampons, added 10 mL of HClO_4_-HNO_3_ mixed acid solution (v/v, 1:2) to digest in the dark at 4°C overnignt, then heated at 120°C, supplementing the mixed acid solution timely. When the solution became clear, it was concentrated to 2 mL. After it was cooled to room temperature, 5 mL of HCl (6 mol·L^-1^) was added. The mixed liquor was heated until the solution became clear, cooled down and accurately diluted into 25 mL with 5% HCl. 1 mL of the solution was transferred to 100 mL volumetric flask and added in 1 mL of 10% potassium ferricyanide, then accurately diluted into 100 mL with 5% HCl as sample solution. The blank sample solution without sSCP was prepared by the same method. The fluorescence intensities of the sample solution and blank sample solution were detected. The selenium contents were calculated according to the regression equation of standard curve.

#### 2.4.2 Infrared spectroscopy analysis

After dried for 4 h in oven, 1 mg of sSCP or SCP was mixed with 200 mg of dried potassium bromide, grinded in the agate mortar and pressed into thin slice. The infrared spectroscopy in wave-number range of 4000–400 cm^-1^ was recorded by FT-IR920 Fourier transform infrared spectrometer (Tianjin Tuopu Instrument Co., Ltd.)

The carbohydrate contents of sSCPs were determined by the phenol-sulfuric acid method.

### 2.5 Comparison of antioxidative activities in vitro

Nine sSCPs and unmodified SCP were diluted with distilled water into five concentration in twofold serially from 1 mg·mL^-1^ to 0.0625 mg·mL^-1^ for use.

#### 2.5.1 DPPH radical-scavenging test

264 test tubes of 10 mL were averagely assigned into 66 groups. The test tubes in ten polysaccharides groups, each five concentrations, were added respectively with 1 mL of polysaccharide solution, in ten polysaccharides control (PC) groups with 1 mL of polysaccharide and 1 mL of anhydrous ethanol, and in one blank control (BC) group with 1mL of anhydrous ethanol. Then the test tubes in polysaccharide groups and BC group were added with 2 mL of DPPH solution, mixed well and reacted for 30 min in 25°C water bath away from light. The absorbance in BC group (A_0_), polysaccharides group (A_1_) and PC group (A_2_) was measured by ultraviolet spectrophotometer (754-type, Shanghai Precision & Scientific Instrument Co., Ltd.) at wavelength of 517 nm. The DPPH radical- scavenging rates were calculated according to the equation: DPPH radical-scavenging rate $=[{\bar{A}}_{0}-({\bar{A}}_{1}-{\bar{A}}_{2})]/{\bar{A}}_{0}\times 100\%$ [21].

#### 2.5.2 Hydroxyl radical-scavenging test

Test 1: 224 test tubes of 10 ml were equally assigned into 56 groups. The test tubes in ten polysaccharides groups, each five concentrations were added respectively with 1 mL polysaccharide solution, in one BC group with 1 mL of distilled water. Then all tubes were added with 2 ml of FeSO_4_·7H_2_O at 9 mmol·L^-1^, 2 mL of salicylic acid at 9 mmol·L^-1^ and mixed well. Finally 2 ml of H_2_O_2_ at 8.8 mmol·L^-1^ was added to start reaction. After the reaction at 37°C for 30 min, the absorbance in BC group (A_0_), and polysaccharides group (A_1_) were measured by ultraviolet spectrophotometers at a wave length of 510 nm. The hydroxyl radical-scavenging rates were calculated according to equation: hydroxyl radical-scavenging rate $=[{\bar{A}}_{0}-({\bar{A}}_{1}-{\bar{A}}_{2})]/{\bar{A}}_{0}\times 100\%$ [22].

Test 2: 224 test tubes of 10 ml were equally assigned into 56 groups. The test tubes in ten polysaccharides groups, each five concentrations, were added respectively with 0.5 mL of polysaccharide solution, in BC group, with 0.5 mL of phosphate buffered (20 mM, pH 7.4,). Then all test tubes were added with 2.5 mL of PBS, 0.5 mL of 2-deoxy-D-ribose (3.75 mM,), 0.5 mL of edathamil disodium (5mM,), 0.5mL of FeCl_3_ (5 mM,), 0.5 mL of H_2_O_2_ (3 mM,) and 0.5 mL of ascorbic acid (5mM). The mixture was incubated at 37°C for 1 h, then 1 mL of mixture was taken, 1 mL of 1% (w/v) trichloroacetic acid and 1 mL of TBA were added, and reacted at 100°C for 20 min. After the mixture was cooled down, the absorbances in BC group (A_0_) and polysaccharides groups (A_1_) were measured with UV/Vis 752 spectrophotometer (JINGHUA, Shanghai, China) at a wave length of 532 nm. The hydroxyl radical-scavenging rates were calculated according to the equation: hydroxyl radical-scavenging rate $=[{\bar{A}}_{0}-({\bar{A}}_{1}-{\bar{A}}_{2})]/{\bar{A}}_{0}\times 100\%$ [23, 24].

#### 2.5.3 ABTS radical-scavenging test

On 96-well culture plate, the wells in ten polysaccharide groups, each five concentrations, were added with 20 μL of polysaccharide solution, 4 wells per concentration, the wells in BC group with 20 μL of PBS. Then all wells were added with 100 μL of ABTS^**·+**^ solution and mixed well. The plates were shaken to react for 6 min in 25°C water bath away from light [25]. The absorbance in BC group (A_0_) and polysaccharides group (A_1_) were immediately measured by ultraviolet spectrophotometers at a wave length of 734 nm. The ABTS^**·+**^ scavenging rates were calculated according to the equation: ABTS^**·+**^ scavenging rate $=[{\bar{A}}_{0}-({\bar{A}}_{1}-{\bar{A}}_{2})]/{\bar{A}}_{0}\times 100\%$.

### 2.6 Comparison of antiglycative activities in vitro

According to the results of free radical-scavenging tests, sSCP_1_ was picked out. Its antiglycative activity was determined by non-enzymatic protein glycation test taking unmodified SCP as control. 27 cell culture bottles were equally assigned into 9 groups. The bottles in BC group were added with 5mL of bovine serum albumin (BSA, 20 mg·mL^-1^) and 10 mL of PBS. The bottles in other groups were added with 5mL of BSA, 5mL of glucose (500 mM) and 5mL of PBS. Then 5mL of sSCP_1_ and SCP at 1, 0.5, 0.25 mg·mL^-1^ were respectively added into the bottles of six polysaccharides groups; 5 mL of aminoguanidine (AG, 1 mg·mL^-1^) was added into the bottles of positive control group (PC); 5mL of PBS was added into the bottles of glycated control group (GC). Then, the mixtures were incubated at 37°C under aseptic conditions [26]. On the seventh, fourteenth, twenty-first days after incubation, the non-enzymatic protein glycation products were determined.

#### 2.6.1 Determination of Amadori product

On seventh days after incubation, 0.5 mL of mixture from each group was mixed with 2 mL of nitro blue tetrozolium reagent (NBT). After incubation for 20 min at room temperature, cold ethanoic acid (w/v: 15%) was added to stop the reaction and the absorbance of reactant was determined using a UV/Vis 752 spectrophotometer (JINGHUA, Shanghai, China) at a wave length of 530nm as the indicator of Amadori products level, and the inhibition percentage of Amadori products was calculated according to the absorbance in BC group (A_0_), polysaccharides groups (A_1_) and glycated control (A_2_) and the equation: inhibition percentage = (*A*_2_ − *A*_1_)/(*A*_2_ − *A*_0_)×100% [27].

#### 2.6.2 Determination of α-dicarbonyl compound

On the fourteenth days after incubation, 0.4 mL of mixture from each group was mixed with 0.2 mL of Girard-T stock solution (500mM) and 3.4 mL of sodium formate (500mM, pH 2.9). After incubation for 1 h at room temperature, the absorbance of reactant was determined using a UV/Vis 752 spectrophotometer at a wave length of 294 nm as the indicator of α-dicarbonyl compounds level, and the inhibition percentage of α-dicarbonyl compounds was calculated according to the absorbance in BC group (A_0_), polysaccharides groups (A_1_) and glycated control (A_2_) and the equation: inhibition percentage = (*A*_2_ − *A*_1_)/(*A*_2_ − *A*_0_)×100% [28].

#### 2.6.3 Determination of AGEs

On the twenty-first days after incubation, 1 mL of mixture from each group was diluted with PBS to10 ml. Their fluorescence intensities (F) in BC group (F_0_), polysaccharides groups (F_1_) and glycated control (F_2_) were measured at 370 nm excitation and 440 nm emission using a TECAN Infinite M200 spectrofluorometer. The relative fluorescence intensities were used as the indicator of AGEs level. The inhibition percentage of AGEs was calculated according to the equation: inhibition percentage = (*F*_2_ − *F*_1_)/(*F*_2_ − *F*_0_)×100% [29].

### 2.7 Comparison of antioxidative activity in vivo

According to the results of test in vitro sSCP_1_ was selected. sSCP_1_ and SCP were diluted into 2 mg·mL^-1^ with distilled water. The diluted preparations were sterilized by pasteurization and detected for endotoxin by pyrogen tests. When the endotoxin content was up to the standard of Chinese Veterinary Pharmacopeia (less than 0.5 EU·mL^-1^) [30], they were stored for the test at 4°C [31].

#### 2.7.1 Experimental animals

According to our previous study [32], one-day-old White Roman chickens (male) which were obtained from Tangquan Poultry Farm and reared in wire cages in the experimental animal house at 37°C with 24 h light in the initial period. The temperature was gradually reduced to the room temperature. The chickens were allowed ad libitum access to water and pathogen-free feed, and commercial starter diet provided by the feed factory of Jiangsu Academy of Agricultural Science.

#### 2.7.2 Experimental design

When the chickens grew to fourteen days of age (average maternal ND-HI antibody titer at 2.8 log2), one hundred and twenty chickens were randomly divided into 4 groups averagely and were vaccinated with ND which vaccine except blank control (BC) group, repeated vaccination at 28 days old. At the same time of each vaccination, the chickens in two polysaccharide groups were intramuscularly injected with 0.5 ml of sSCP_1_ and SCP (2 mg·ml^-1^) respectively. The chickens in BC and vaccination control (VC) groups were intramuscularly injected with the equal volume of physiological saline. On days 7 (D_7_), 14 (D_14_), 21 (D_21_) and 28 (D_28_) after the first injection, the blood of four chickens randomly from each group were sampled for the determination of serum contents of CAT, SOD and MDA.

The whole experimental procedures were performed in strict accordance with internationally accepted principles and Chinese legislation and the animal experiment project has been approved by the Nanjing Agricultural University Animal Care Committee with the guide for the use and care of laboratory animals. During whole experiment session, each process was rigorously in accordance with the regulation of animal protection committee to minimize the suffering and injury. After the experiment was completed, the chickens were killed humanely by euthanasia of CO_2_.

### 2.8 Statistical analysis

The antioxidant index data were expressed as means ± SD. Duncan’s multiple range test was used to analyze the difference among groups. Significant difference between means was considered at *P* < 0.05.

## Results

### 3.1 The modification conditions, yields and contents of selenium and carbohydrate of sSCPs

The modification conditions, yields and contents of selenium and carbohydrate of sSCPs are listed in Table 1. The yield of sSCP_2_ was the highest (54.87%), the next were sSCP_5_, sSCP_3_ and sSCP_6_. The selenium content of sSCP_3_ was the highest (10.35 mg·g^-1^), the next were sSCP_4_, sSCP_1_ and sSCP_2_. The carbohydrate content of sSCP_1_ was the highest (56.83%), next were sSCP_4_, sSCP_5_ and sSCP_2_.

### 3.2 The infrared spectra of SCP and sSCP

**Fig 1:**
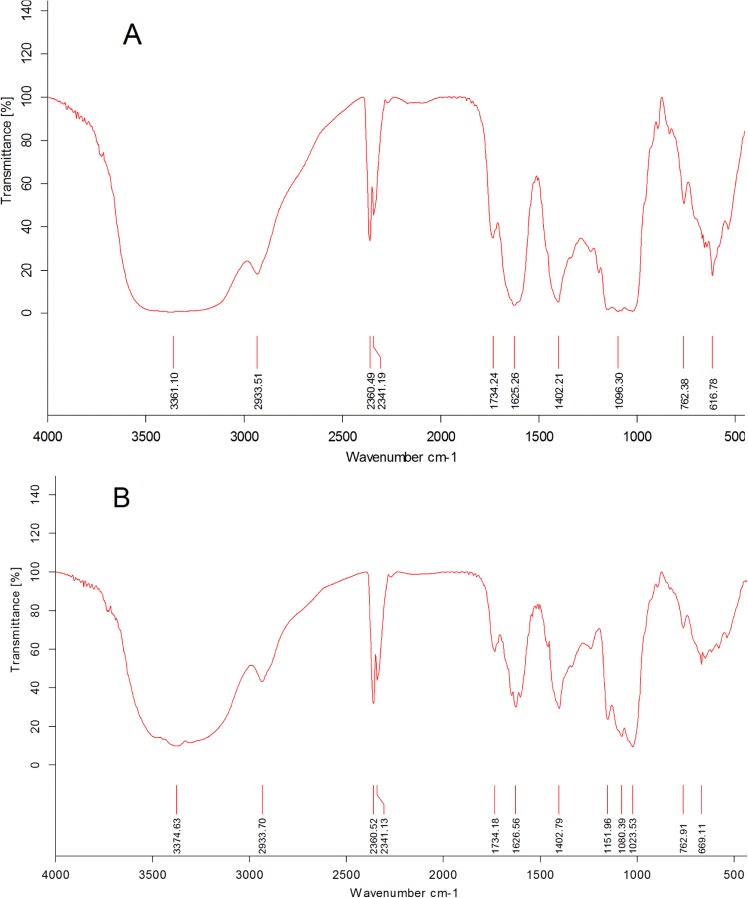
Infrared spectra of SCP (A) and SCP (B).

The FT-IR spectra of SCP and sSCP in 4000–400 cm^-1^ are illustrated in Fig 1. In the spectra of SCP and sSCP, there were three characteristic vibration peaks of polysaccharides. One appeared in 3600–3200 cm^-1^ corresponding to the hydroxyl stretching vibration. The second appeared in 1400–1000 cm^-1^corresponding to C–O–C stretching vibration. The third appeared in the 2932 cm^-1^corresponding to methyl C-H stretching vibration. As compared with the spectrum of SCP, the spectrum of sSCP presented two characteristic absorption bands, one appeared at 1023.53 cm^-1^ describing a symmetrical O–Se–O stretching vibration (*v*_*as*_, O–Se–O, 1010–1040 cm^-1^) and another at 669.11 cm^-1^ describing an asymmetrical Se–O–C stretching vibration (*v*, Se–O–C, 600–700 cm^-1^) (Fig 1B), which indicated that sSCP was successfully modified in selenylation and mainly in the form of RSeO_3_R^r^ [33].

### 3.3 Antioxidant activities in vitro of sSCP and SCP

#### 3.3.1 Changes of DPPH radical-scavenging rate

**Fig 2:**
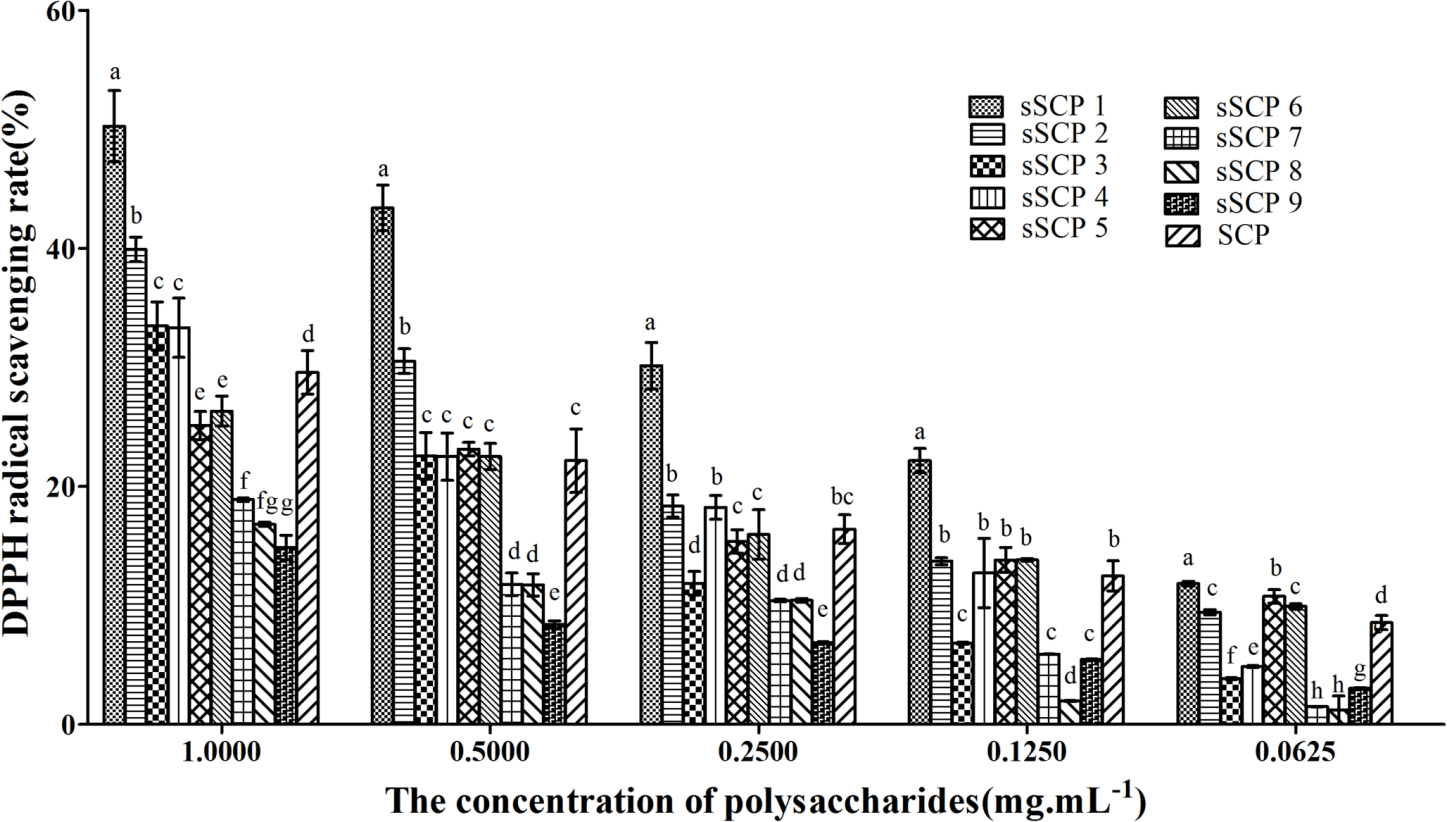
The DPPH radical-scavenging rates in each group at five concentrations. Bars in same concentration without same superscripts (a–h) differ significantly (*P* < 0.05).

The DPPH radical-scavenging rates in each group at five concentrations are illustrated in Fig 2. The DPPH radical-scavenging rates in each group at five concentrations presented dose dependent manner. At the concentration of 1 mg·mL^-1^, the DPPH radical-scavenging rates in sSCP_1_-sSCP_4_ groups were significantly higher than that in SCP group (*P*<0.05). The scavenging rates in sSCP_1_ and sSCP_2_ at 0.5 mg·mL^-1^ groups were significantly higher than that in SCP group (*P*<0.05). The scavenging rates in sSCP_1_ at 0.25, 0.125 mg·mL^-1^ groups were significantly higher than that in SCP groups at the same concentration (*P*<0.05). The scavenging rates in sSCP_1_-sSCP_2_, sSCP_5_-sSCP_6_ at 0.0625 mg·mL^-1^ groups were significantly higher than that in SCP group (*P*<0.05). The scavenging rates in sSCP_1_ group were the highest at all concentrations.

#### 3.3.2 Changes of hydroxyl radical-scavenging rate

**Fig 3:**
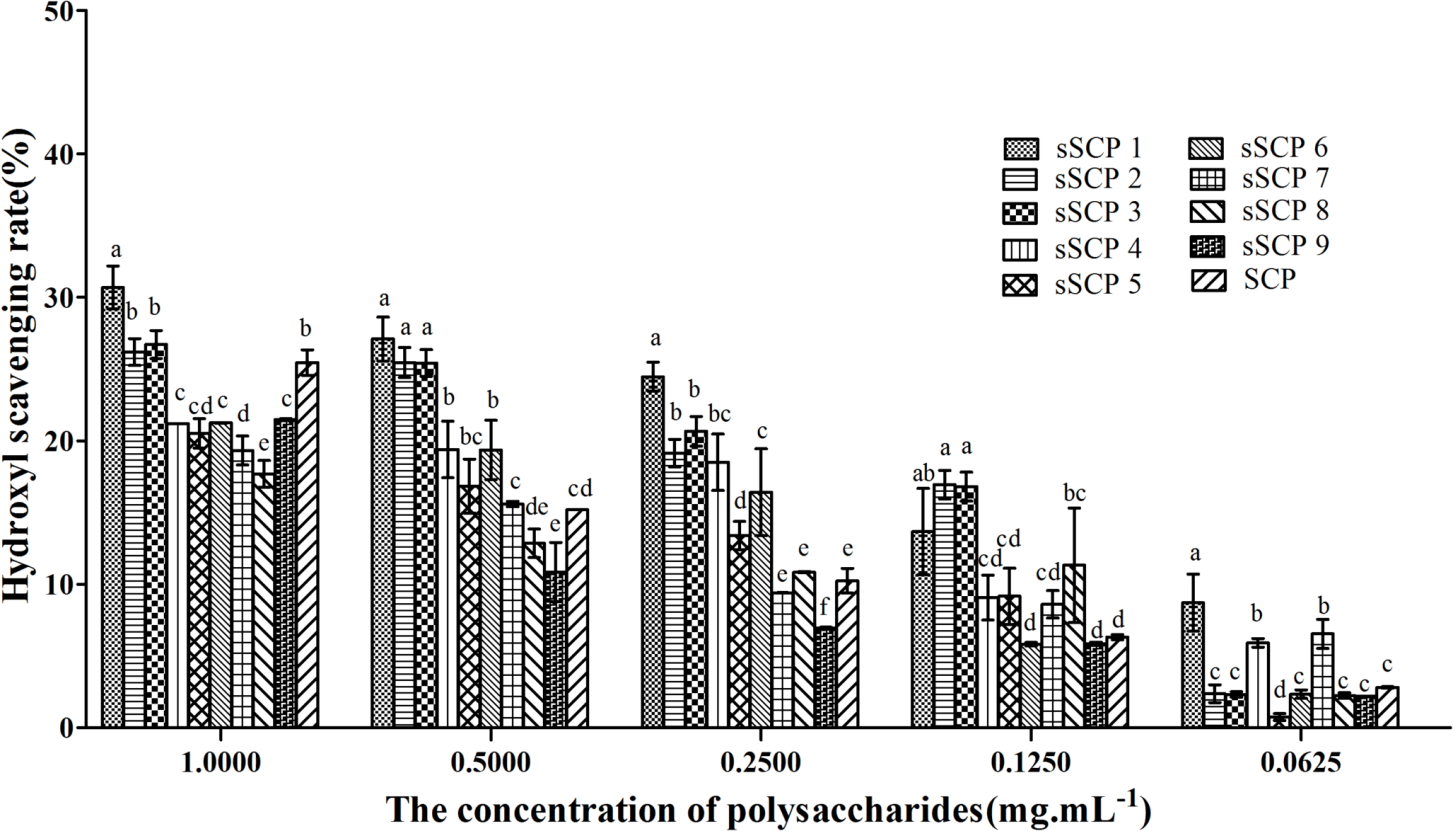
The hydroxyl radical scavenging rates in each group at five concentrations. Bars in same concentration without same superscripts (a–f) differ significantly (*P* < 0.05).

**Fig 4:**
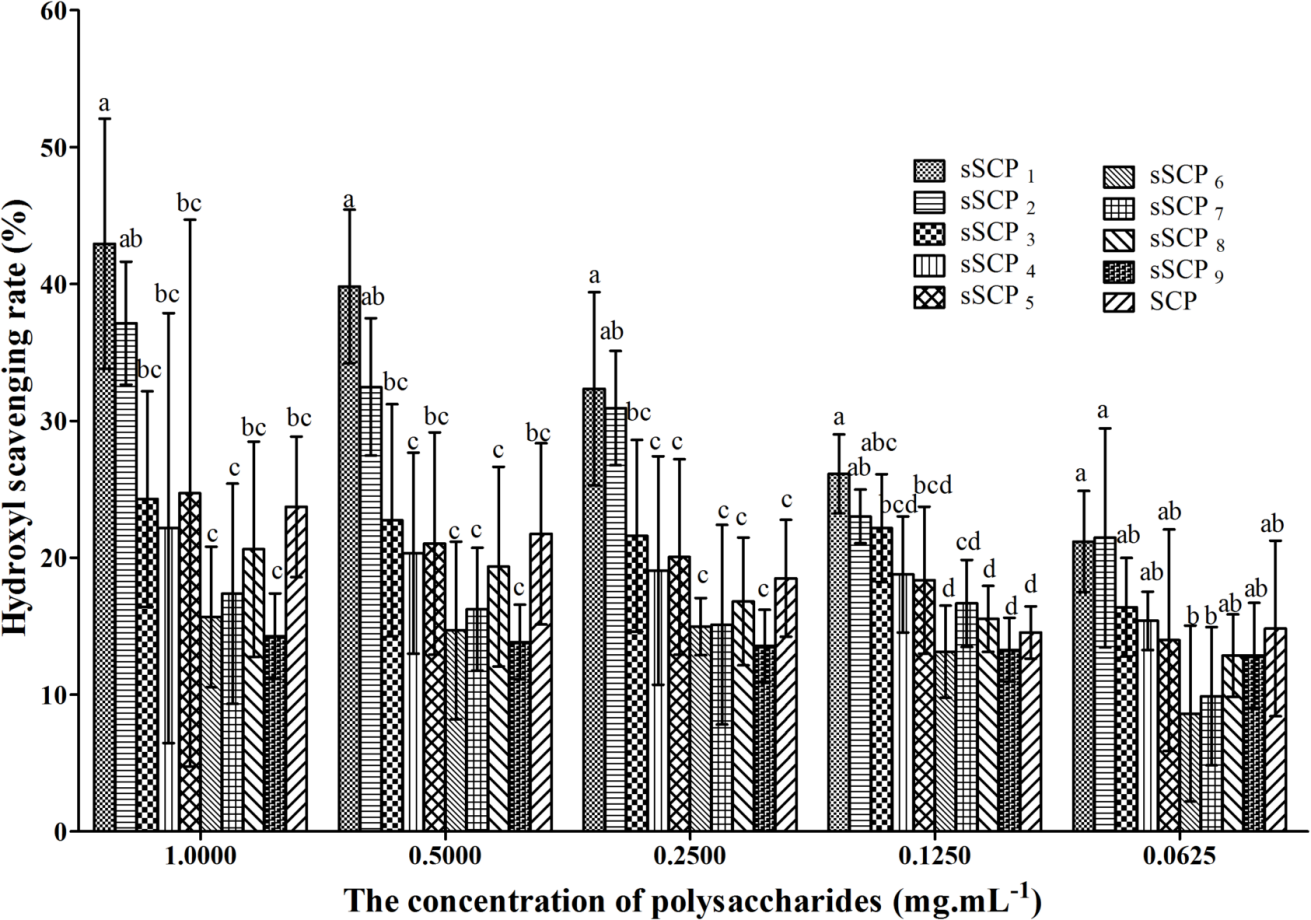
The hydroxyl radical-scavenging rates in each group at five concentrations (TBARS). Bars in same concentration without same superscripts (a–d) differ significantly (P < 0.05).

The hydroxyl radical-scavenging rates in each group at five concentrations are illustrated in Figs 3 and 4. The hydroxyl radical-scavenging rates in each group at five concentrations presented dose dependent manner. In test 1, the hydroxyl radical-scavenging rate in sSCP_1_ at 1 mg·mL^-1^ group, in sSCP_1_–sSCP_4_ and sSCP_6_ at 0.5 mg·mL^-1^ groups, in sSCP_1_–sSCP_6_ at 0.25 mg·mL^-1^ groups, in sSCP_1_–sSCP_3_ and sSCP_8_ at 0.125 mg·mL^-1^ groups and in sSCP_1_, sSCP_4_ and sSCP_7_ at 0.0625 mg·mL^-1^ groups were significantly higher than that in SCP at same concentration group respectively (*P*<0.05). The scavenging rates in sSCP_1_ at all concentrations except 0.125 mg·mL^-1^ group were the highest.

In test 2, the hydroxyl radical-scavenging rate in sSCP_1_ at 1 and 0.5 mg·mL^-1^ groups, in sSCP_1_–sSCP_2_ at 0.25 mg·mL^-1^ groups and in sSCP_1_–sSCP_3_ at 0.125 mg·mL^-1^ groups were significantly higher than that in SCP at same concentration group respectively (*P*<0.05). The scavenging rates in sSCP_1_ at all concentrations except 0.0625 mg·mL^-1^ group were the highest.

#### 3.3.3 Changes of ABTS radical-scavenging rate

**Fig 5:**
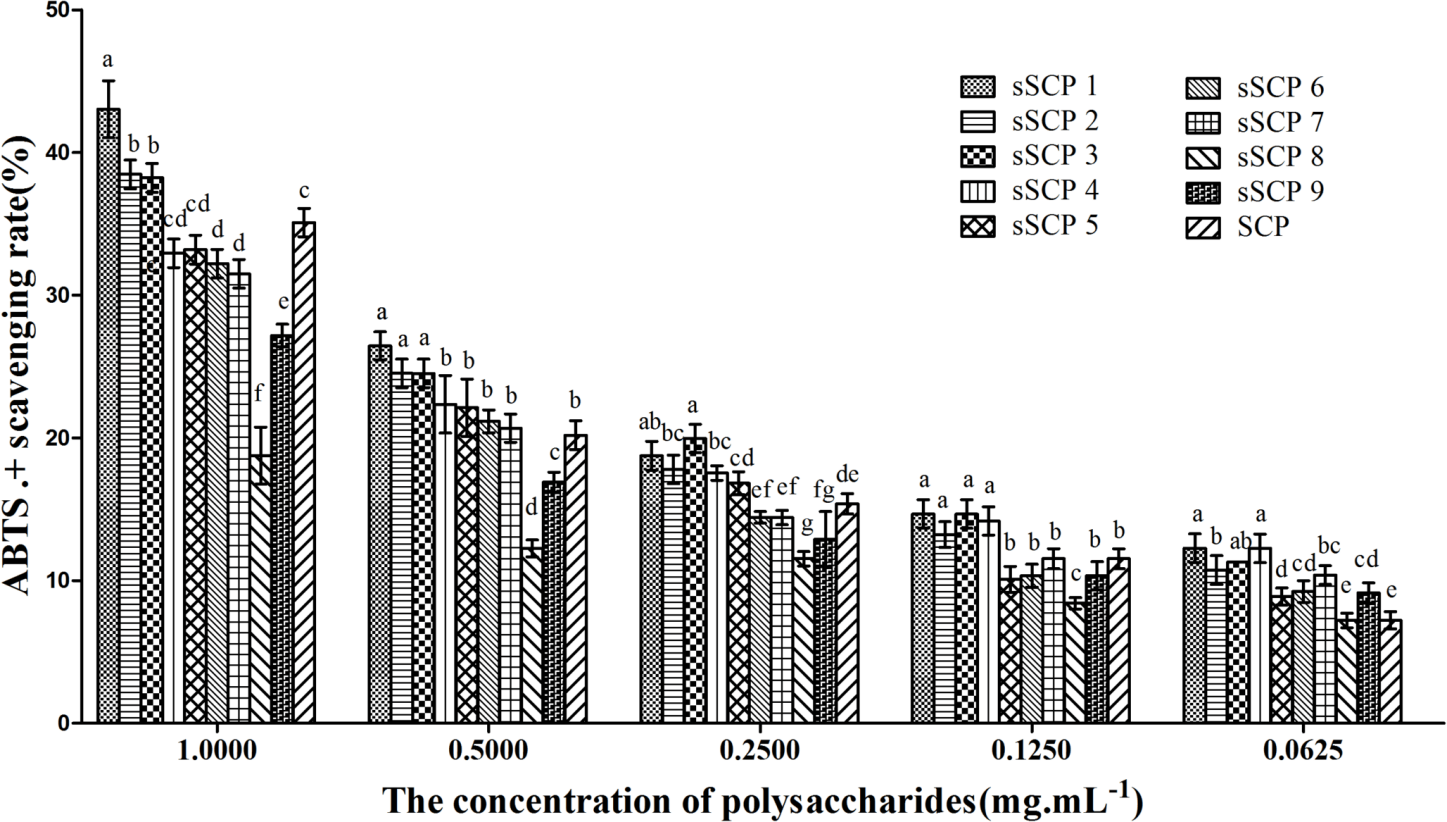
The ABTS *radical-*scavenging rates in each group at five concentrations. Bars in same concentration without same superscripts (a–g) differ significantly (*P* < 0.05).

The ABTS^**·+**^ radical-scavenging rates in each group at five concentrations are illustrated in Fig 5. The ABTS radical-scavenging rates in each group at five concentrations presented dose dependent manner. The scavenging rates in sSCP_1_–sSCP_3_ at 1, 0.5 mg·mL^-1^ groups were significantly higher than that in SCP groups at the same concentration (*P*<0.05). The scavenging rate in sSCP_1_–sSCP_4_ at 0.25, 0.125 mg·mL^-1^ groups were significantly higher than that in SCP groups at the same concentration (*P*<0.05). The scavenging rate in sSCP_1_–sSCP_7_ and sSCP_9_ at 0.0625 mg·mL^-1^ groups were significantly higher than that in SCP group (*P*<0.05). The scavenging rates in sSCP_1_ at all concentrations except 0.25 mg·mL^-1^ group were the highest.

### 3.4 Antiglycative activity of sSCP_1_

**Fig 6:**
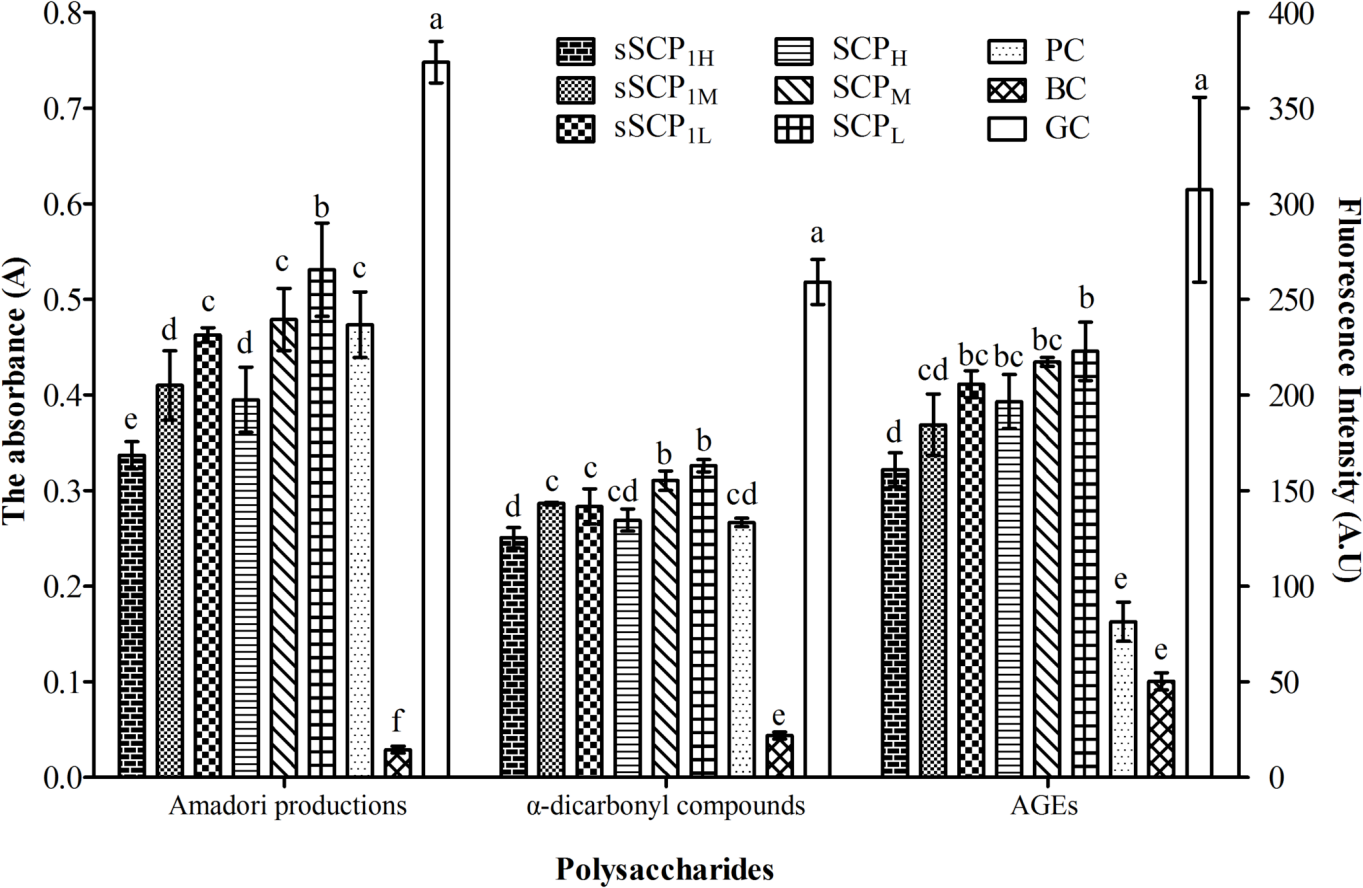
The absorbance of Amadori products and α-dicarbonyl compounds and the relative fluorescence intensities of AGEs in each group. [sSCP_1L_ = BSA+Glu+sSCP_1_ (0.25mg·mL^-1^); sSCP_1M_ = BSA+Glu+sSCP_1_ (0.5mg·mL^-1^); sSCP_1H_ = BSA+Glu+sSCP_1_ (1mg·mL^-1^); SCP _L_ = BSA+Glu+SCP (0.25mg·mL^-1^); SCP_M_ = BSA+Glu+SCP (0.5mg·mL^-1^); SCP_H_ = BSA+Glu+SCP (1mg·mL^-1^); PC = BSA+Glu+AG; BC = BSA+PBS; GC = BSA+Glu]. Bars in same concentration without same superscripts (a–f) differ significantly (*P* < 0.05).

**Fig 7:**
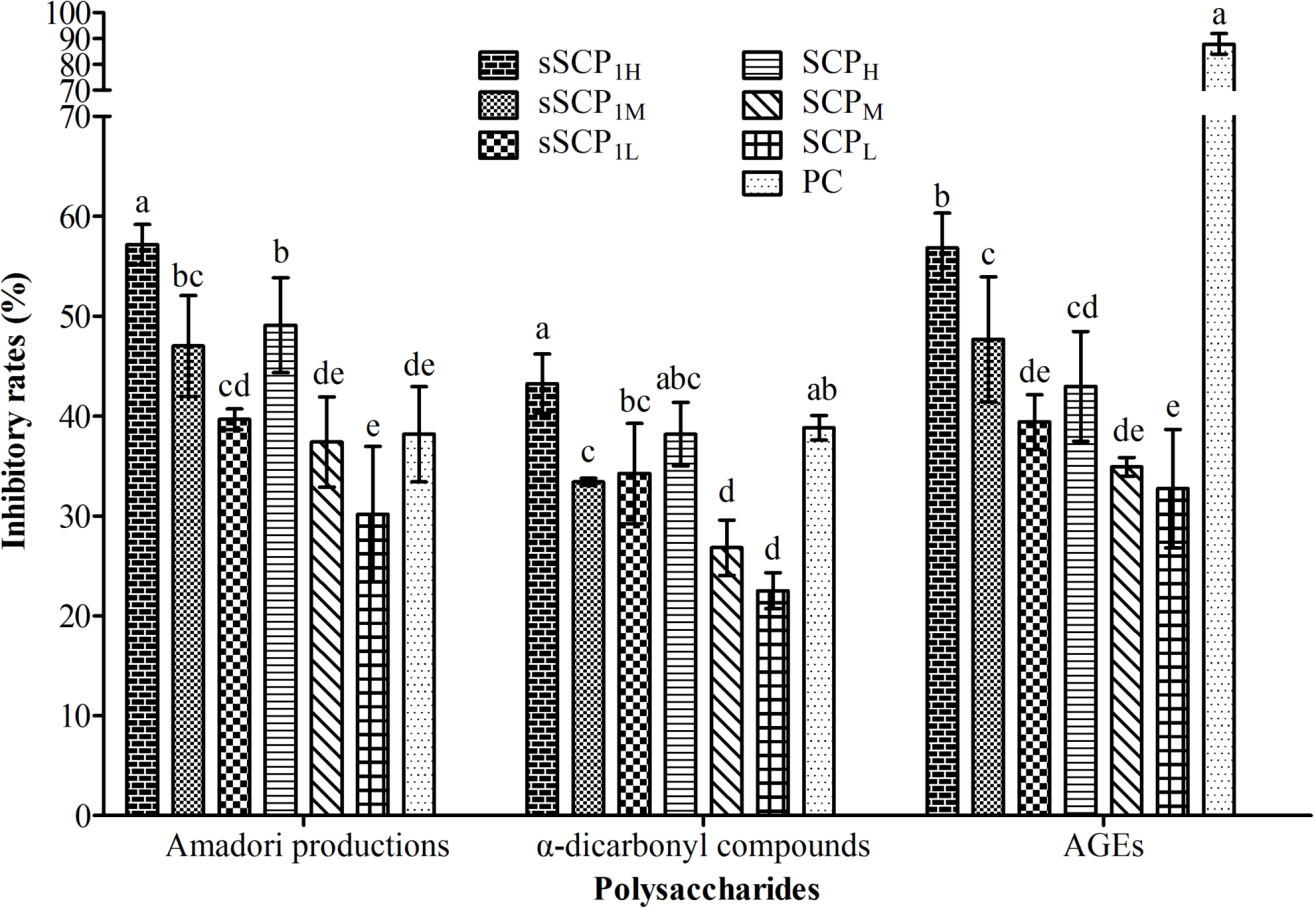
The inhibitory rates of Amadori products, α-dicarbonyl compounds and AGEs in each group. Bars in same concentration without same superscripts (a–e) differ significantly (*P* < 0.05).

The absorbance and inhibitory rates of Amadori products, α-dicarbonyl compounds and the relative fluorescence intensities of AGEs and inhibitory rates in each group are respectively illustrated in Fig 6 and Fig 7.

#### 3.4.1 Change of Amadori products

The absorbance and inhibitory rates in three concentration groups of sSCP_1_ and SCP presented dose dependent manner. The absorbance in sSCP_1_ at 1 and 0.5 mg·mL^-1^ groups were respectively significantly lower than that of SCP at same concentration group (*P*<0.05). The absorbance in sSCP_1_ at 1 and 0.5 mg·mL^-1^ groups were significantly lower than that in PC group (*P*<0.05). The absorbance in six polysaccharide groups and PC group were significantly lower than that in GC group (*P*<0.05).

The inhibitory rates in sSCP_1_ at three concentrations groups were respectively significantly higher than that in SCP at same concentration group (*P*<0.05). The inhibitory rates in sSCP_1_ at 1 and 0.5 mg·mL^-1^ groups were significantly higher than that in PC group (*P*<0.05). The inhibitory rate in sSCP_1_ at 1 mg·mL^-1^ group was the highest.

#### 3.4.2 Change of α-dicarbonyl compounds

The absorbance and inhibitory rates in three concentration groups of sSCP_1_ and sSCP presented dose dependent manner. The absorbance in sSCP_1_ at 0.5 and 0.25 mg·mL^-1^ groups were respectively significantly lower than that in SCP at same concentration group (*P*<0.05). The absorbance of six polysaccharide groups and PC group were significantly lower than that in GC group (*P*<0.05).

The inhibitory rates in sSCP_1_ at 1 and 0.5 mg·mL^-1^ groups were respectively significantly higher than that in SCP at same concentration group (*P*<0.05). The inhibitory rate in sSCP_1_ at 1 mg·mL^-1^ group was the highest and numerically higher than that in PC group (*P*>0.05).

#### 3.4.3 Change of AGEs

The relative fluorescence intensities and inhibitory rates in three concentration groups of sSCP_1_ and sSCP presented dose dependent manner. The fluorescence intensities in six polysaccharide groups and PC group were significantly lower than that in GC group (*P*<0.05). The fluorescence intensity in sSCP_1_ at 1 mg·mL^-1^ group was the lowest among six polysaccharide groups, and significantly lower than those in SCP group at three concentrations (*P*<0.05).

The inhibitory rates in sSCP_1_ at 1 and 0.5 mg·mL^-1^ groups were respectively significantly higher than that in SCP at same concentration group (*P*<0.05). The inhibitory rate in sSCP_1_ at 1 mg·mL^-1^ group was the highest among six polysaccharide groups.

### 3.5 Antioxidant activities in vivo of sSCP and SCP

#### 3.5.1 Changes of serum CAT activity

**Fig 8:**
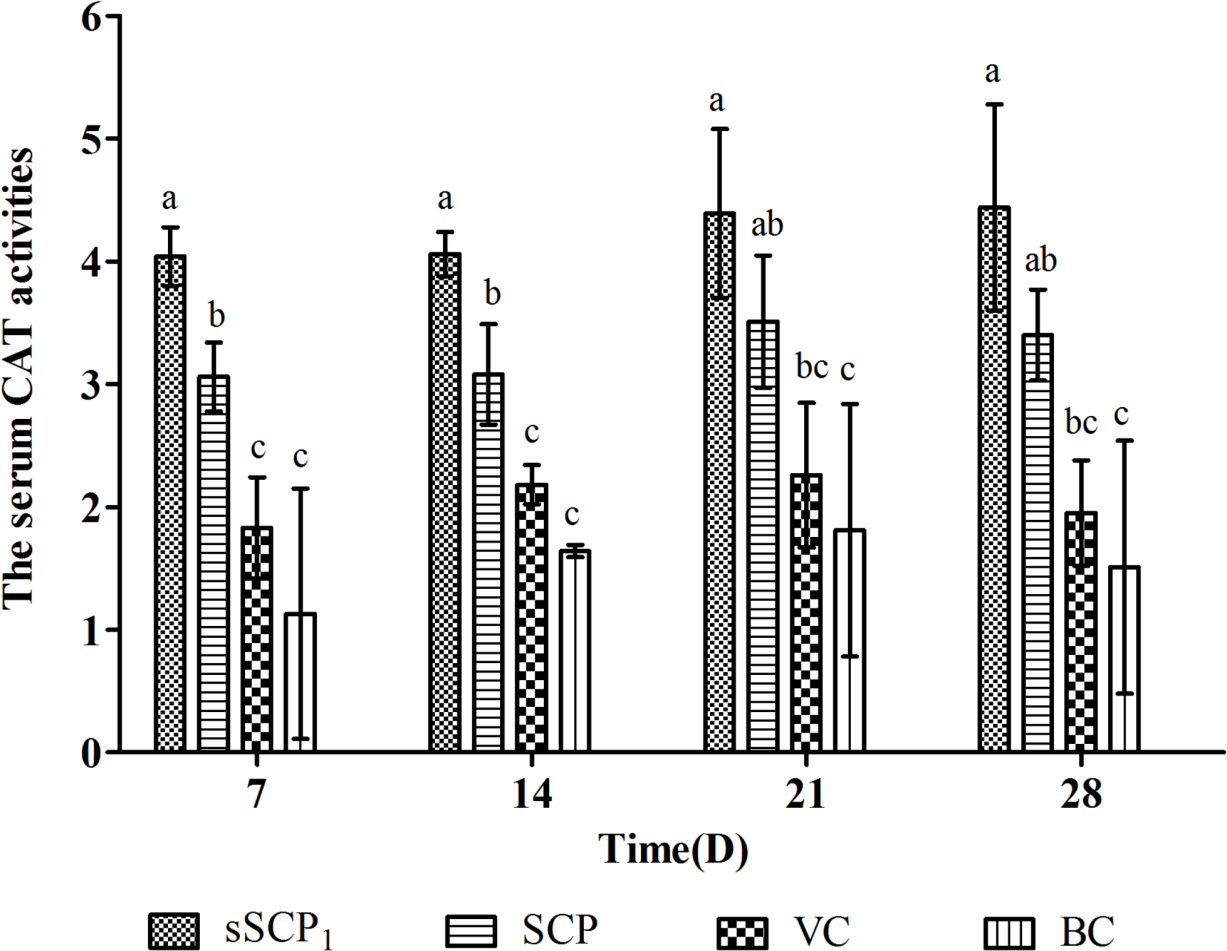
The serum CAT activities in each group. Bars in same time without the same superscripts (a–c) differ significantly (*P* < 0.05).

The serum CAT activities in each group are listed in Fig 8. At all time points after injection, the serum CAT activities in sSCP_1_ group were the highest, significantly higher than those in VC and BC groups (*P*<0.05), on D_7_–D_14_ significantly higher than those in SCP group and on D_21_–D_28_ numerically higher than those in SCP group (*P*>0.05). The serum CAT activities in SCP group on D_7_–D_14_ were significantly higher than those in VC and BC group (*P*<0.05) on D_21_–D_28_ significantly higher than that in BC group (*P*<0.05) and numerically higher than that in VC group (*P*>0.05).

#### 3.5.2 Changes of serum SOD activity

**Fig 9:**
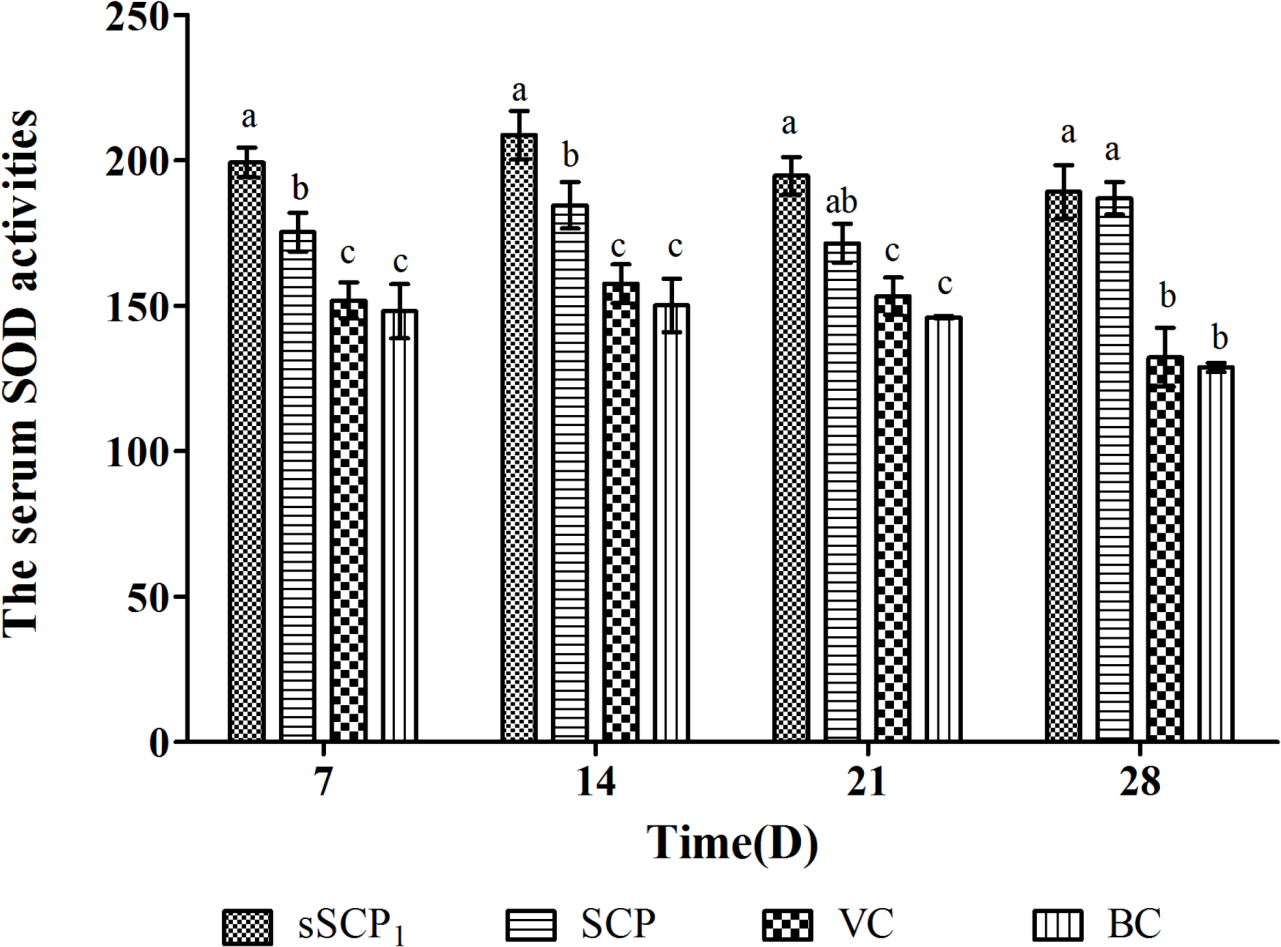
The serum SOD activities in each group. Bars in same time without the same superscripts (a–c) differ significantly (*P* < 0.05).

The serum SOD activities in each group are listed in Fig 9. At all time points after injection, the serum SOD activities in sSCP_1_ and SCP groups were significantly higher than those in VC and BC groups (*P*<0.05), in sSCP_1_ group were the highest, on D_7_–D_14_ significantly higher than those in SCP group (*P*<0.05) and on D_21_–D_28_ numerically higher than those in SCP group (*P*>0.05).

#### 3.5.3 Changes of serum MDA contents

**Fig 10:**
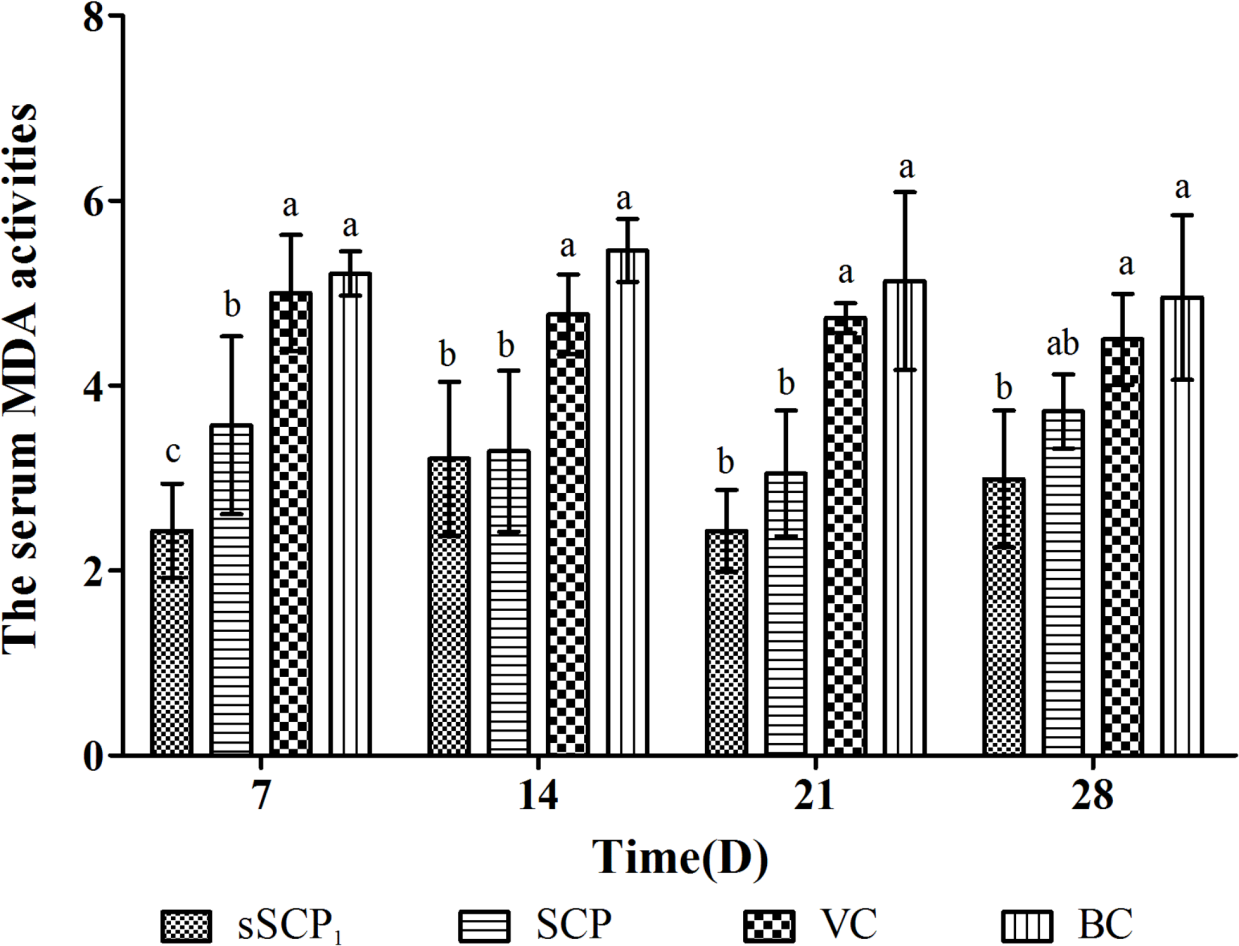
The serum MDA activities in each group. Bars in same time without the same superscripts (a–c) differ significantly (*P* < 0.05).

The serum MDA contents in each group are listed in Fig 10. At all time points after injection, the serum MDA contents in sSCP_1_ group were the lowest, significantly lower than those in VC group and BC group (*P*<0.05), on D_7_ significantly lower than that in SCP group (*P*<0.05) and on D_14_–D_28_ numerically lower than those in SCP group (*P*>0.05). The serum MDA contents in SCP group on D_7_–D_21_ were significantly lower than those in VC group and BC group (*P*<0.05) and on D_28_ numerically lower than those in VC group and BC group (*P*>0.05).

## Discussion

Owing to different antioxidant components having different scavenging activities against various ROS, there are several methods to evaluate the antioxidative activity in vitro of a compound, such as DPPH radical-scavenging test, hydroxyl radical-scavenging test, ABTS radical-scavenging test and so on [16]. DPPH is a stable free-radical compound and is widely used to evaluate the ability of antioxidants to scavenge radicals. Hydroxyl radical is one of the most reactive and dangerous free radicals among ROS and is mainly responsible for the oxidative injury.

The scavenging rate for these free radicals can reflect the antioxidant activity of a polysaccharide [5, 34]. In this study, the antioxidant activities in vitro of nine sSCPs and unmodified SCP at five concentrations were compared. The results showed that the scavenging rates for DPPH radical, hydroxyl radical and ABTS radical of all polysaccharide presented dose dependent manner. The scavenging rates in sSCPs at some concentration groups were significantly higher than that of unmodified SCP, and in sSCP_1_ at five concentration groups were the highest. These results indicate that the antioxidant activities of these sSCPs were significantly higher than that of unmodified SCP, selenylation modification can significantly enhance the antioxidant activity in vitro of SCP, sSCP_1_ has the highest efficacy.

According to the results of free radical-scavenging test, sSCP_1_ was selected and further validated its antioxidant activity in vivo. SOD and CAT are the protective enzyme and free radical scavenger in organism. They play an important role in resisting oxidative damage [35]. CAT is a hemoprotein in all aerobic cells and can scavenge surplus H_2_O_2_ to maintain cells in stabile internal environment and normal activity. The results of this test showed that at all time points after injection, the serum CAT activities in sSCP_1_ group were the highest, significantly higher than those in VC and BC groups, on D_7_–D_14_ significantly higher than those in SCP group and on D_21_–D_28_ numerically higher than that in SCP group, while in SCP group on D_7_–D_14_ were significantly higher than those in VC and BC group, and on D_21_–D_28_ significantly higher than that in BC group and numerically higher than that in VC group. These indicate that unmodified SCP has antioxidant activity, while the activity of SCP is much stronger and selenylation modification could further improve the antioxidant activity of SCP. Some studies have confirmed that selenium and polysaccharide can synergistically enhance the scavenging effect for hydroxyl radical [36].

SOD is a scavenger of peroxide anion radicals, can suppress the initiation of lipid peroxidation caused by free radicals and plays an important role in maintaining the balance of oxidation and antioxidation. It can clear O^-2^ and protects cells from damage. Serum SOD content can reflect the antioxidant ability of organism [37]. This test showed that at all time points after injection, the serum SOD activities in sSCP_1_ and SCP groups were significantly higher than those in VC and BC groups, in sSCP_1_ group were the highest, on D_7_–D_14_ significantly and on D_21_–D_28_ numerically higher than those in unmodified SCP group. These indicated that both sSCP_1_ and SCP posses antioxidant activity, the activity of sSCP_1_ is significantly stronger than that of unmodified SCP, and selenylation modification can further improve the antioxidant activity of SCP in vivo. Our previous study proved that selenylation modification could significantly improve the antioxidant activity of lycium barbarum polysaccharide [38].

MDA is the metabolic product of lipid peroxidation, its content in serum or tissue can reflect the injury degrees of lipid peroxidation and the severity of oxidation [39]. This test showed that at all time points after injection the serum MDA contents in sSCP_1_ group were the lowest, significantly lower than those in VC group and BC group, on D_7_ significantly and on D_14_–D_28_ numerically lower than those in SCP group, while in SCP group were significantly or numerically lower than those in VC group and BC group. These results also confirm that selenylation modification can further improve the antioxidant activity of SCP in vivo. Other research found that the effective components of Se-enriched lactobacillus could reduce MDA content of liver in mice [40].

Glycation and oxidative stress are closely linked, which is involved in the pathogenesis of many age-related chronic diseases [41]. In organism, the nonenzymatic glycation known as Maillard reaction is easily happened between the reducing sugar and the amino groups of proteins, lipids and nucleic acids. During the process of reaction, it is started with the reversible formation of Schiff base, which undergoes a rearrangement to form stable Amadori products, finally generate cross-linked, irreversible and fluorescent compounds called as advanced glycation end products (AGEs) [29.] One of the mechanisms which AGEs exert cytotoxicity is binding to their receptors (RAGE). ROS is generated in the processes of early and the advanced glycation and it induces oxidative stress through the reaction with RAGE [42].

NBT could be reduced by Amadori products to generate a coloured reactant. α-dicarbonyl compound, glycation intermediate, is easily reacted with amino to generate AGEs, and AGEs is characterized by fluorescent. According to these principles, nonenzymatic protein glycation and the antiglycative activity of selenizing polysaccharide can be determined [43]. The results of this determination showed that sSCP1 and SCP could significantly inhibit nonenzymatic protein glycation during three periods of reaction. In high dose groups of sSCP1 and SCP, the inhibitory rates for Amadori products were significantly higher than that in PC group, and the inhibitory rates for α-dicarbonyl compounds were not significantly different from that in PC group. The inhibitory rates for three glycation products in sSCP_1_ at three concentrations groups were almost respectively significantly higher than that in SCP at same concentration group. These confirmed that sSCP_1_ and SCP possess stronger antiglycative activity, the action of sSCP_1_ is significantly stronger than that of SCP, and selenylation modification can further improve the antioxidant activity of SCP.

There are several strategies to defense oxidative stress such as preventing DNA damage, inhibiting lipid peroxidation and initiation of oxidative chain reaction, and activating and synthesizing antioxidants to scavenge free radicals. The antioxidant defence includes enzymes (superoxide dismutase, catalase and glutathione peroxidase) and non-enzymes systems (glutathione, ascorbic acid and α-tocopherol) [44], therefore, the ideal antioxidant can activate the enzyme system, inhibit the production of free radicals or scavenge free radicals. Our results showed that sSCP_1_ could not only scavenge DPPH·, hydroxyl radical and ABTS^**·+**^ and antiglycation in vitro, but also elevated SOD and CAT activities and decreased MDA content of serum in vivo, it is a kind of idealer antioxidant. Some reports consider that to defense oxidative stress may be by activating specific, genetic or metabolic upstream signal pathways or down-regulating oxidative stress and cell apoptotic signal pathway to synthetize protein isoforms and lipids which are less sensitive to oxidation [45, 46].

It is believed that selenizing polysaccharide has diploid or higher pharmacological activities as compared with selenium or polysaccharide, the antioxidant activity of selenizing polysaccharide was related to its selenium content and the polysaccharide content, but it was not that the higher content of selenium or polysaccharide, the higher its activity [37]. This also is proved in this experiment. Among the nine sSCPs, sSCP_1_ presented strongest antioxidant activities, its selenium and carbohydrate contents were arranged respectively in third and first place. While sSCP_3_ with the highest selenium content and unmodified SCP with the highest polysaccharide content, their antioxidant activity were significantly lower than that of sSCP_1_. From this it can be seen that the activity of selenizing polysaccharide depends on the synergism of its optimal selenium content with carbohydrate content. The further characterization of *Schisandra chinensis* polysaccharide (SCP) was modified in selenylation would be investigated in the future.

In conclusion, selenylation modification can significantly enhance the antioxidant activity in vitro and vivo of SCP, sSCP_1_ possesses best efficacy and can be as a component drug of polysaccharide antioxidants, its modification conditions can be as the optimal conditions to enhance antioxidant activity of SCP, that is 200 mg of sodium selenite per 500 mg of SCP, the reaction temperature of 50°C and the reaction time of 6 h.
